# Efficacy of acupuncture treatment in visual field defect: A case report

**DOI:** 10.1097/MD.0000000000042088

**Published:** 2025-08-01

**Authors:** Li-Li Xie, Kai-Liang Luo, Xiao-Ping Cheng, Qun-Lin Chen, Lan Lv, Li-Qiong Zhan, Le-Wen Chen, Xin-Yuan Chen, Di Wu, Yue-Zhu Zhou

**Affiliations:** a Department of Rehabilitation Medicine, The First Affiliated Hospital, Fujian Medical University, Fuzhou, China; b Department of Radiology, The First Affiliated Hospital, Fujian Medical University, Fuzhou, China; c National Regional Medical Center, Binhai Campus of the First Affiliated Hospital, Fujian Medical University, Fuzhou, China.

**Keywords:** acupuncture, brain injury, central–peripheral–central, closed-loop rehabilitation, neural plasticity, neurorehabilitation, visual field defect

## Abstract

**Rationale:**

Visual field defect refers to an impairment of the visual field resulting from damage to the retina, optic nerve, or brain. Visual field defect has a significant impact on the daily life of the patient. There are few clinical trials of acupuncture for visual field loss and even fewer trials of peripheral-central acupuncture for visual field loss. We report a case of visual field loss due to trauma.

**Patient concerns:**

Despite undergoing oral and maxillofacial surgery and visual rehabilitation training at our hospital, the patient continued to have low light sensitivity, significant visual field fluctuations, and poor eye movement coordination in his right eye.

**Diagnoses:**

The patient was diagnosed with a visual field defect.

**Interventions:**

According to the clinical experience of Professor Jin Rui, a renowned acupuncturist in China, we selected “three eye-needling” and “three brain-needling” techniques to treat the patient. As a traditional neuroregulatory rehabilitation technique, this treatment scheme conforms to the treatment paradigm of “central-peripheral-central closed-loop rehabilitation” through the combination of central and peripheral intervention.

**Outcomes:**

After 6 months of treatment, patients reported improvement in symptoms and we observed significant improvements in the Humphrey perimetry results of the patient’s right eye. These findings suggest a considerable enhancement in the visual field. At the same time, the quality of life of patients also improved.

**Lessons:**

The findings demonstrate the feasibility and effectiveness of “peripheral-central” bidirectional acupuncture in treating visual field defects in this case. This study demonstrated the application of the “central-peripheral-central closed-loop rehabilitation” mode in clinical practice through the application of traditional Chinese medicine characteristic therapies “three eye-needling” and “three brain-needling.”

## 1. Introduction

Visual field defect (VFD) refers to an impairment of the visual field resulting from damage to the retina, optic nerve, or brain. The etiology of VFD is complex and diverse, encompassing conditions such as optic nerve atrophy, glaucoma, other peripheral neuropathies, stroke, brain tumors, and other central neuropathies. These conditions can compromise optic nerve function, leading to vision loss, visual field defects, and other visual impairments.^[1]^ VFD significantly impacts a patient’s daily life and imposes a substantial burden on families and society. In recent years, the treatment methods for VFD have gradually diversified, including visual restoration training, noninvasive brain stimulation, and medications that enhance blood flow. The recovery of the visual field can be achieved through various means, such as visual restoration training, noninvasive brain stimulation, or medications that enhance blood flow. However, these treatments may have drawbacks such as high costs, drug side effects, unclear mechanisms, and the need for long-term adherence. In contrast, acupuncture, as a natural therapy, offers the advantages of being simple to perform and having minimal side effects, making it a potential option for VFD treatment. In 2016, Professor Jia proposed the closed-loop rehabilitation concept of “central-peripheral-central” for the first time, and it has been widely used in the field of rehabilitation.^[2]^ This model aims to stimulate and activate the brain region to regulate the excitability of the cerebral cortex through central intervention, and regulate the center through peripheral stimulation feedback to promote brain function remodeling and nerve reinnervation, which can play a synergistic effect and further promote the functional recovery of patients. Research has shown that acupuncture, by stimulating peripheral nerve endings, can induce the transmission of neural impulses, which in turn feedback to the central nervous system, modulating its excitability and functional state.^[3]^ For instance, acupuncture stimulation of periocular acupoints (such as BL-1 and ST-1) can enhance local blood circulation, improve peripheral nerve function, and activate visual centers through neural feedback mechanisms, thereby promoting the repair of visual pathways.^[4]^ Additionally, acupuncture facilitates neuroplastic changes and the reorganization of functional networks in the central nervous system by regulating neurovascular coupling mechanisms and the release of neurotrophic factors.^[2,5]^ These mechanisms collectively contribute to the physiological foundation of a “peripheral-central” closed-loop rehabilitation. Several studies have demonstrated that acupuncture is effective for peripheral nerve injury and brain diseases. The potential mechanisms underlying its effects involve nervous system remodeling during nerve repair.^[4,6]^ In particular, acupuncture promotes neuroplastic changes by modulating the interaction between the central and peripheral nervous systems, thereby improving visual function.^[2]^

Although the application of acupuncture in the treatment of VFD has gradually garnered attention, related research remains relatively limited. Early studies primarily focused on the efficacy of acupuncture for conditions such as glaucoma and optic atrophy. For example, research by Wu^[4]^ demonstrated that acupuncture, through stimulation of periocular acupoints, could significantly improve visual field defects and visual acuity in glaucoma patients. Additionally, studies by Yang et al^[3]^ further confirmed that acupuncture promotes the repair and remodeling of visual pathways by modulating the excitability of the central nervous system, thereby improving visual function in VFD patients. However, most of these studies are small-scale or single-center trials, lacking support from large-scale randomized controlled trials.

With the development of the “peripheral-central” closed-loop rehabilitation theory and the in-depth study of the neural mechanism of acupuncture, we try to adopt “peripheral-central” bidirectional adjustment acupuncture and moxibustion rehabilitation treatment plan to improve the curative effect of this disease. Here, we report a case of a VFD patient with peripheral nerve injury, whose symptoms significantly after treatment with “peripheral-central” bidirectional acupuncture. By integrating the “three-eye needling” and “three-brain needling” techniques, we have further validated the feasibility and efficacy of acupuncture in the treatment of VFD.

## 2. Case presentation

**Figure 1.**
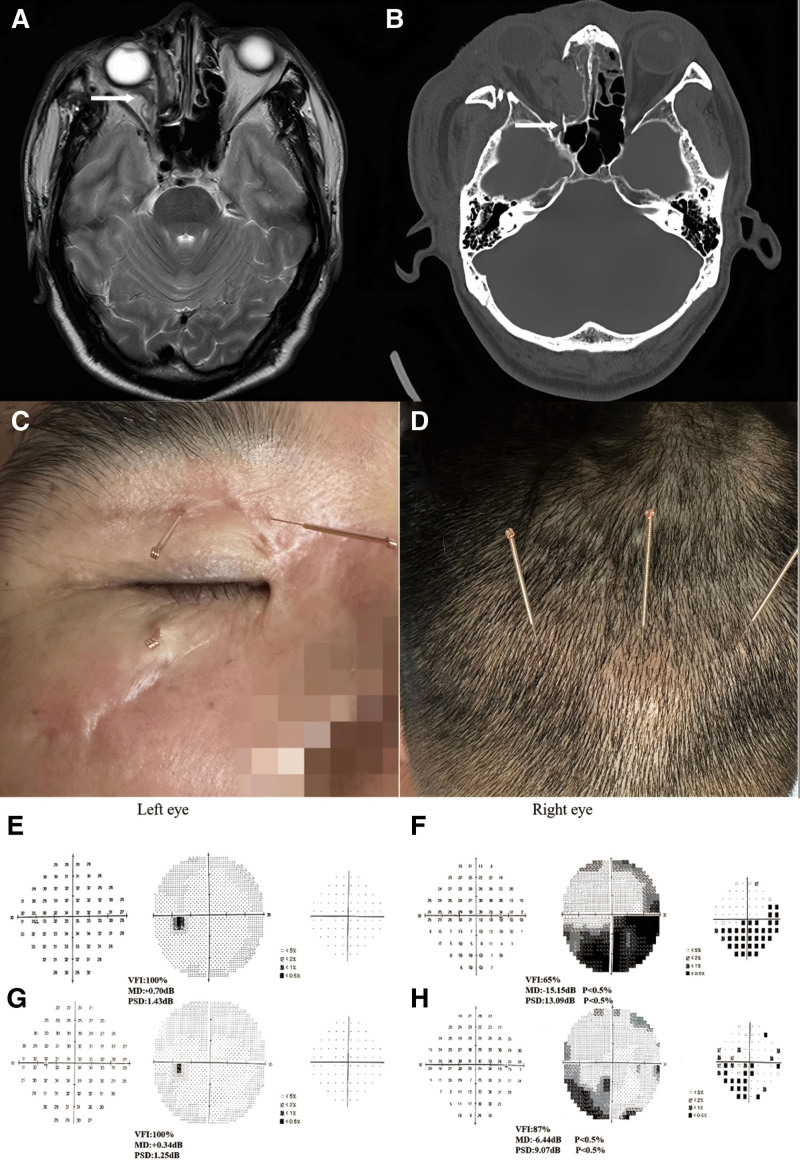
MR and CT findings showing a fracture of the optic canal, injure and swelling of the optic nerve on the right side (A and B). Humphrey visual field examination results in the patient (C and D). First examination of the patient’s binoculus (E and F); second examination of the patient’s binoculus (G and H). CT = computed tomography.

A 46-year-old male patient working on a fishing boat experienced an accident when a bolt cover exploded, sending metal fragments into his head. This caused pain, bleeding on the right side of his face, and blurred vision in his right eye. The magnetic resonance imaging and computed tomography scan revealed an injure of optic nerve and a fracture of the optic canal (Fig. 1A and B). Humphrey perimetry of the right eye indicated VFI at 65%, MD at ‐15.15 dB, PSD at 13.09 dB, and a visual acuity (logMAR) of 0.7, confirming a visual field defect (Fig. 1E and F, File S1, Supplemental Digital Content, https://links.lww.com/MD/O643). Despite undergoing oral and maxillofacial surgery and visual rehabilitation training at our hospital, the patient continued to experience low light sensitivity, significant visual field fluctuations, and poor eye movement coordination in his right eye. At the same time, the assessment of the patient’s quality of life and mental state found that the patient’s SF36 score was 48 points, indicating poor health. The score of HRSD scale was 28 points, indicating that the patient was moderately depressed. After observing the patient’s condition for half a year, he received acupuncture treatment at our suggestion. The patient understood this study in accordance with the provisions of the Declaration of Helsinki (1964) and provided written consent. The study was approved by the Ethics Committee of the First Affiliated Hospital of Fujian Medical University (MRCTA, ECFAH [2015]084).

According to the clinical experience of Professor Jin Rui, a renowned acupuncturist in China, we selected “three eye-needling” and “three brain-needling” techniques to treat the patient.^[6]^ The “three eye-needling” are as follows: Eye I needle is positioned 2.5 mm above the Jingming point, and the Chengshi point is located by Eye II needle. The 3 eye-needling technique targets the pupil directly upward, aligning with the superior orbital margin between the eyeballs.

The “three brain-needling” points include the Naohu point and the Naokong point. After disinfecting the skin, we used a disposable acupuncture needle with a diameter of 0.30 mm and a length of 40 mm. We instructed the patient to lie on their back and close their eyes for the “three eye-needling” procedure (Fig. 1C). The practitioner gently stabilized the eyeball laterally with the left hand and inserted the needle vertically to a depth of 25 mm to 30 mm with the right hand. To avoid puncturing blood vessels and causing hematomas, we did not twist, lift, or insert the needle further. Instead, we gently scraped the needle handle with the thumbnail. After needle insertion, we pressed the needle hole gently with a dry cotton ball to prevent bleeding. For the “three brain-needling,” we performed flat needling to a depth of 20 to 30 mm (Fig. 1D). Each treatment session lasted 30 minutes, and we conducted the treatment 3 times a week for 2 months, constituting one course. The entire treatment spanned 3 courses.

After 6 months of treatment, patients reported improvement in symptoms and at the same time we observed significant improvements in the Humphrey perimetry results of the patient’s right eye. The VFI increased to 87%, while the MD decreased to ‐6.44 dB and the PSD reduced to 9.07 dB compared to the initial values. These findings suggest a considerable enhancement in the visual field, with a reduction in the degree of visual field defect. Additionally, the visual acuity (logMAR) improved to 0.22, indicating a substantial overall improvement in visual function (Table 1, Fig. 1G and H, File S1, Supplemental Digital Content, https://links.lww.com/MD/O643). At the same time, the patient’s SF36 score was 88, indicating that the patient’s health status had changed to good. The HRSD scale score was 12, indicating that the patient had changed to mild depression.

**Table 1:** Results of two-time Humphrey visual field examinations and visual acuity in the patient’s left and right eye.

	Visual field index	Mean deviation (dB)	Pattern standard deviation (dB)	Visual acuity (logMAR)
**Left eye**				
First time	100%	0.7	1.43	0
Second time	100%	0.34	1.25	0
Percentage of change	0%	–	–	–
**Right eye**				
First time	65%	‐15.15	13.09	0.70
Second time	87%	‐6.44	9.07	0.22
Percentage of change	22%	–	–	–

Percentage of change = VFI (second time) ‐ VFI (first time).

## 3. Discussion

Acupuncture represents a frequently utilized conservative therapy for ophthalmic disorders. This case study affirms the efficacy of “three eye-needling’’ and “three brain-needling’’ in ameliorating visual field defects and enhancing visual acuity subsequent to optic nerve damage. The intervention involving “three eye-needling’’ coupled with “three brain-needling’’ constitutes a traditional neuromodulation rehabilitation protocol that integrates central and peripheral aspects, aligning with the rehabilitation paradigm of “central-peripheral-central closed-loop rehabilitation.”^[6]^

The region surrounding the eyes contains 3 acupuncture points referred to as “three eye-needling.” Among these, eye Ⅱ needle and eye Ⅲ needle are associated with the oculomotor nerve, eye channel, and blood vessels. Acupuncture at these points can directly enhance blood circulation and regulate nerve function in the surrounding tissues. Additionally, stimulation of nerve endings around the eye promotes the release of impulses, enhancing the flexibility and coordination of local muscle movements. Furthermore, the special peripheral sensory afferent mode of acupuncture can adjust the excitability of neurons in the neural reflex pathway, so as to realize the functional reorganization and functional compensation of the cerebral cortex, so as to restore the normal regulatory.^[7]^ Moreover, numerous studies indicate the good treatment efficacy of acupuncture in treating neurological impairments resulting from peripheral nerve injuries, and its underlying mechanism likely involves nervous system remodeling during nerve repair.^[3]^ Acupuncture stimulation of peripheral nerves induces feedback stimulation to the central nervous system, facilitating improved connectivity between the central and peripheral nervous systems, ultimately enhancing visual function.

Different from traditional acupuncture, head acupuncture is based on the functional positioning of the cerebral cortex to accurately select head points or specific target areas for stimulation, which not only provides a theoretical basis for the combination of acupuncture and brain science research, but also enriches the methods and types of central intervention as a modern acupuncture therapy.^[8]^ The “three brain-needling” is based on the “head needling” theory situated at the junction of the cerebellum and the occipital lobe of the cerebral cortex at the back of the head, directly stimulate the visual central nervous system. This stimulation enhances blood circulation in the occipital visual cortex,^[9]^ facilitates functional repair of visual nerve cells,^[3]^ and promotes the connection and remodeling of the brain’s co-functional network.^[10]^ Studies have shown that neuronal networks of the brain can “amplify’’ residual vision through neuroplasticity changes of local and global functional connectivity by activating, modulating and strengthening residual visual signals.^[11]^ Consequently, there is a strengthening of the direct link between the central nervous system and the peripheral nerves, leading to improved visual function. Thus, combining the “three brain-needling” points with “three eye-needling” and matching points before and after can play the role of “peripheral–central” bidirectional regulation.

Although presenting satisfactory outcomes obtained in this study, there are some limitations in our study. Firstly, due to the single-case nature of this study, we were unable to perform an unblinded intervention and lacked a control intervention. Secondly, the effects of acupuncture are combined with the patient’s natural recovery at the present time, so the effects of each different treatment are indiscernible. Thus, further studies in large groups are needed to verify the effects.

## 4. Conclusion

The findings demonstrate the feasibility and effectiveness of “peripheral–central” bidirectional acupuncture in treating visual field defects in this case. Acupuncture at “three eye-needling” and “three brain-needling” points accelerates nerve regeneration and repair at both peripheral and central levels, thereby activating visual pathways and ameliorating visual field defects as well as patients’ quality of life. This study demonstrated the application of the “central-peripheral-central closed-loop rehabilitation” mode in clinical practice through the application of traditional Chinese medicine characteristic therapies “three eye-needling” and “three brain-needling.” This acupuncture method not only embodies the holistic view and meridian theory of traditional Chinese medicine, but also the perfect embodiment of the rehabilitation concept of integrated Chinese and Western medicine. Although presenting satisfactory outcomes obtained in this study, there are some limitations in our study.

## Acknowledgments

The authors would like to thank the kind patients, families, and caregivers who participated in this research.

## Author contributions

**Conceptualization:** Kai-Liang Luo, Xiao-Ping Cheng, Yue-Zhu Zhou.

**Data curation:** Di Wu.

**Project administration:** Xin-Yuan Chen.

**Resources:** Li-Qiong Zhan, Lan Lv.

**Software:** Le-Wen Chen.

**Visualization:** Kai-Liang Luo, Qun-Lin Chen.

**Writing – original draft:** Li-Li Xie.

**Writing – review & editing:** Li-Li Xie .

## Supplementary Material


