# Supplementary material for: Efficacy of acupuncture treatment in visual field defect: A case report
Source: Medicine (Baltimore). 2025 Aug 1;104(31):e42088. doi: 10.1097/MD.0000000000042088 (PMC12323948; doi:10.1097/MD.0000000000042088)
Supplement: Supplementary file 1 [file medi-104-e42088-s001.pdf]

Single Field Analysis

Eye Right

Name chuangzhenlong

DOB 1974-09-03

ID 19546

Central 30-2 Threshold Test

Fixation Monitor Gaze/Blind Spot
Fixation Target Central
Fixation Losses 6/15 xx
False POS Errors 19 % xx
False NEG Errors 29 %
Test Duration 08:09

Fovea OFF

Stimulus III White
Background 31.5 ASB
Strategy SITA-Fast

Pupil Diameter 3.2 mm
Visual Acuity
Rx DS DC X

Date 2022-09-17
Time [illegible]
Age 48

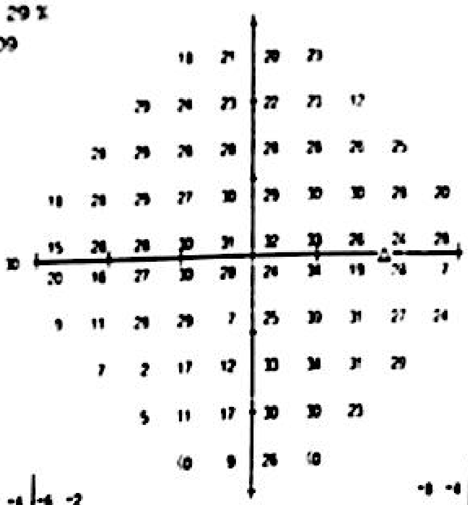

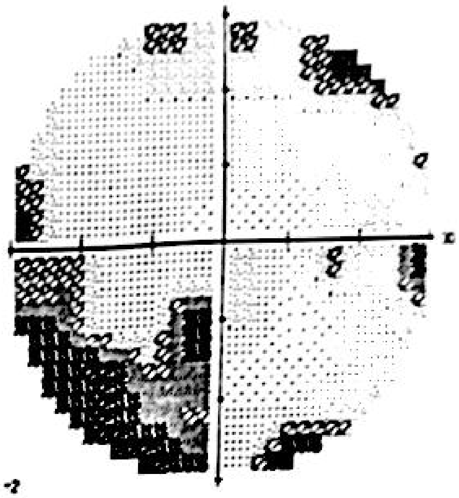

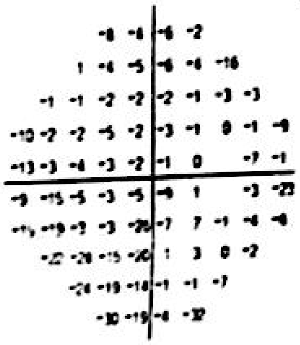

Total Deviation

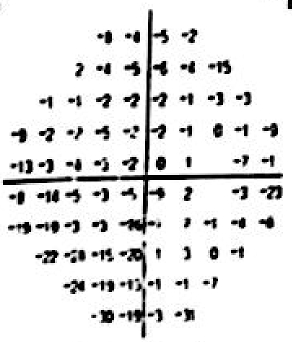

Pattern Deviation

*** Excessive High False Positives ***

GHT
Outside normal limits

VFI 87%

MD -6.44 dB P < 0.5%
PSD 9.07 dB P < 0.5%

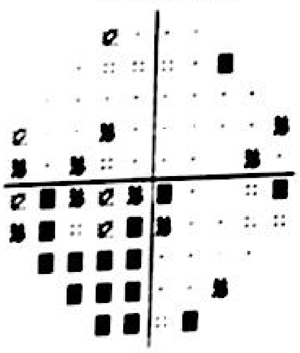

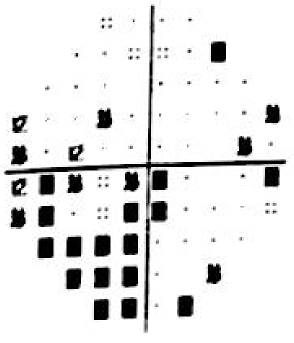

:: < 5%
< 2%
< 1%
< 0.5%

First Affiliated Hospital Of Fujian Medical University

Single Field Analysis

Name: zhuangzhenlong	Eye: Right
ID: 19546	DOB: 1974-09-03

Central 30-2 Threshold Test

Fixation Monitor: Gaze/Blind Spot
Fixation Target: Central
Fixation Losses: 2/15
False POS Errors: 27 % xx
False NEG Errors: 10 %
Test Duration: 08:18

Fovea: OFF

Stimulus: III, White
Background: 31.5 ASB
Strategy: SITA-Fast

Pupil Diameter: 4.2 mm
Visual Acuity:
RX: DS DC X

Date: 2022-03-09
Time: 8:39 AM
Age: 47

			15	21	13	8			
		23	22	25	22	22	19		
	24	27	25	25	28	26	23	20	
26	24	25	28	27	30	30	26	13	15
24	26	26	29	30	30	29	34	12	2
25	23	26	30	21	10	<0	<0	<0	<0
8	15	7	13	7	<0	12	<0	<0	1
	17	5	<0	5	0	11	4	1	
		0	<0	<0	<0	7	<0		
			6	<0	<0	7			

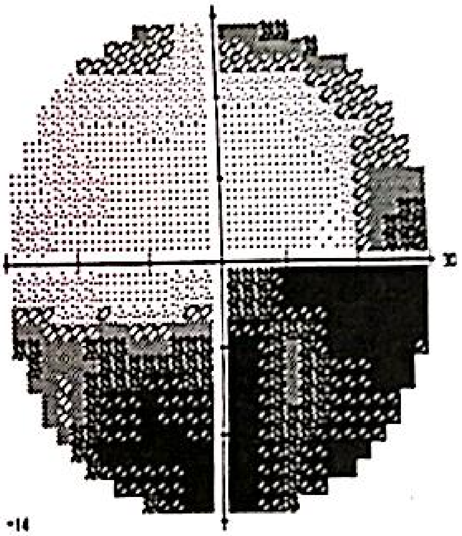

			-11	-5	-12	-17			
		-5	-6	-3	-6	-6	-8		
	-5	-3	-6	-5	-2	-4	-6	-9	
-1	-6	-6	-4	-6	-2	-1	-5	-17	-15
-4	-5	-6	-4	-3	-3	-4		-19	-28
-4	-7	-6	-4	-12	-23	-35		-33	-32
-20	-15	-25	-19	-26	-35	-20	-33	-33	-29
	-13	-26	-33	-26	-32	-20	-27	-29	
		-29	-32	-32	-32	-24	-32		
			-22	-31	-31	-22			

Total Deviation

			-8	-2	-9	-14			
		-2	-3	0	-2	-2	-5		
	-1	0	-2	-1	1	-1	-3	-6	
2	-3	-3	-1	-2	1	2	-1	-14	-11
-1	-1	-3	-1	0	1	-1		-16	-25
0	-4	-3	0	-9	-20	-31		-30	-29
-17	-12	-22	-16	-23	-32	-17	-30	-29	-26
	-9	-23	-30	-23	-29	-17	-24	-26	
		-25	-29	-29	-29	-20	-29		
			-19	-27	-28	-19			

Pattern Deviation

*** Excessive High False Positives ***

GHT
Outside normal limits

VFI 65%

MD -15.15 dB P < 0.5%
PSD 13.09 dB P < 0.5%

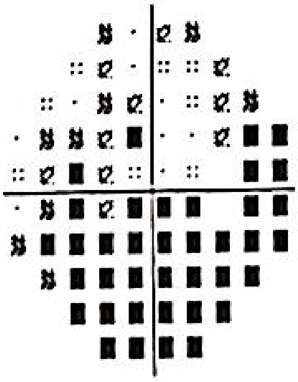

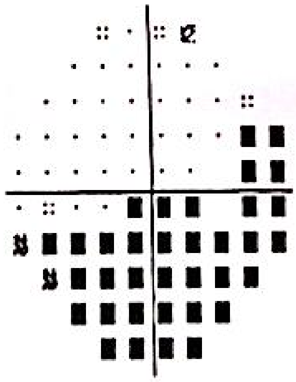

:: < 5%
▨ < 2%
▩ < 1%
■ < 0.5%

First Affiliated Hospital Of
Fujian Medical University

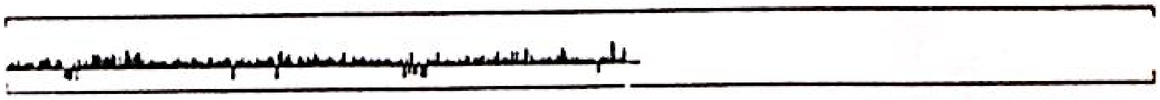

© 2007 Carl Zeiss Meditec
HFA II 750-13392-4.2.2/4.2.2

Single Field Analysis

Eye: Left

Name: zhuangzhenlong

DOB: 1974-[illegible]

ID: 19546

Central 30-2 Threshold Test

Fixation Monitor: Gaze/Blind Spot
Fixation Target: Central
Fixation Losses: 4/11 xx
False POS Errors: 8 %
False NEG Errors: 0 %
Test Duration: 04:01

Fovea: OFF

Stimulus: III, White
Background: 31.5 ASB
Strategy: SITA-Fast

Pupil Diameter: 3.3 mm
Visual Acuity:
RX DS DC X

Date: 2022-[illegible]
Time: 8:49 AM
Age: 47

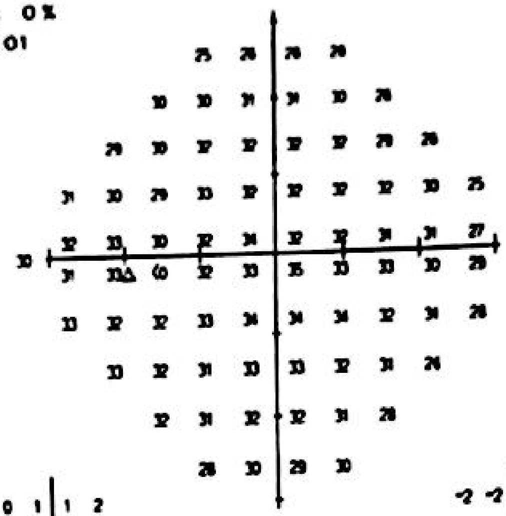

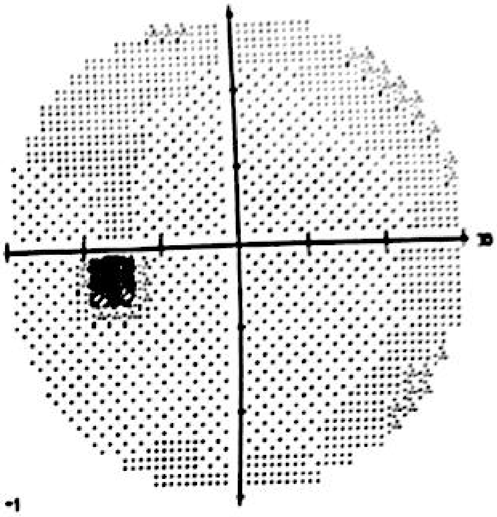

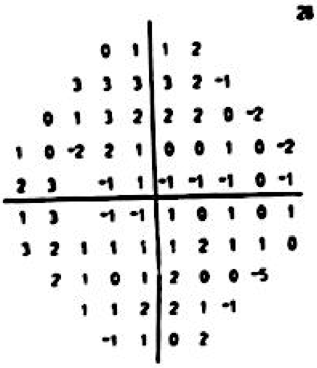

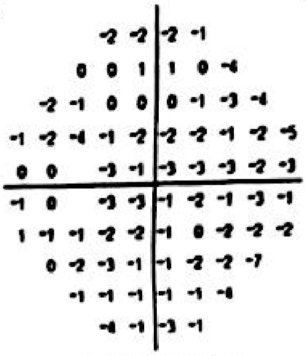

*** Low Test Reliability ***

GHT
Within normal limits

VFI 100%

MD +0.70 dB
PSD 1.43 dB

Total Deviation

Pattern Deviation

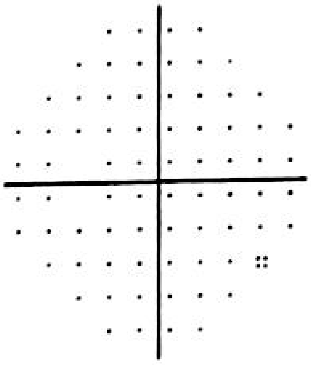

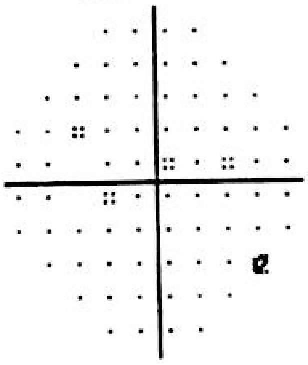

:: < 5%
< 2%
< 1%
■ < 0.5%

First Affiliated Hospital Of
Fujian Medical University

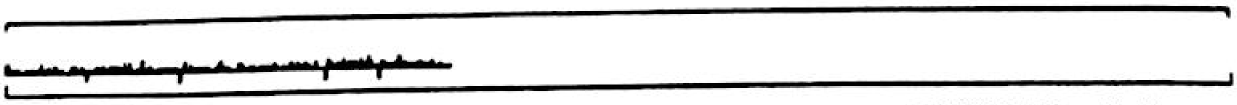

© 2007 Carl Zeiss Meditec
HFA II 750-13392-4.2.2/4.2.2

Single Field Analysis — Eye: Left

Name: Zhuangzhenlong — DOB: 1974-09-03

ID: [illegible]1812

Central 30-2 Threshold Test

Fixation Monitor: Gaze/Blind Spot	Stimulus: III, White	Pupil Diameter: 2.8 mm	Date: 2022-09-17
Fixation Target: Central	Background: 31.5 ASB	Visual Acuity:	Time: 8:05 AM
Fixation Losses: 0/11	Strategy: SITA-Fast	RX: DS DC X	Age: 48
False POS Errors: 0 %			
False NEG Errors: 0 %			
Test Duration: 03:29			
Fovea: OFF			

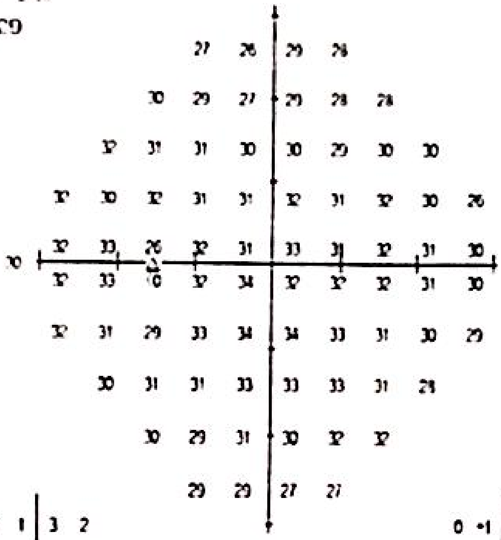

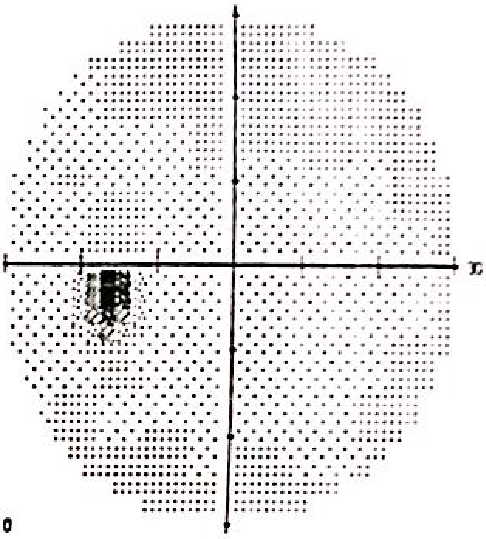

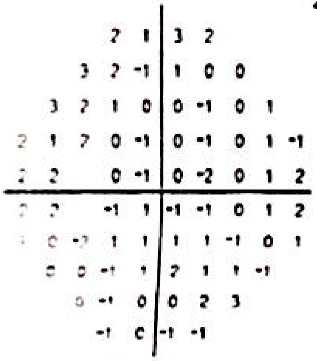

Total Deviation

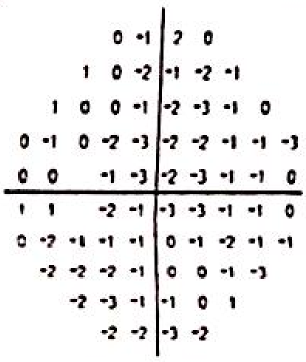

Pattern Deviation

GHT

Within normal limits

VFI 100%

MD +0.34 dB

PSD 1.25 dB

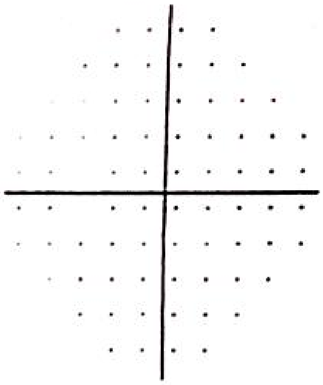

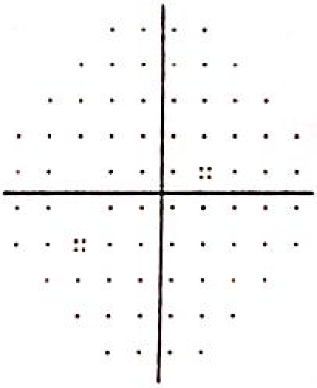

:: < 5%

⁑ < 2%

▓ < 1%

■ < 0.5%

First Affiliated Hospital Of Fujian Medical University

© 2007 Carl Zeiss Meditec

HFA II 750-13392-4.2.2/4.2.2
